# Discovery of Isograndidentatin D, a Novel Phenolic Glycoside, and Anti-*Helicobacter pylori* Phenolics from *Salix koreensis* Twigs

**DOI:** 10.3390/plants13243603

**Published:** 2024-12-23

**Authors:** Yoon Seo Jang, Dong-Min Kang, Yoon-Joo Ko, Moon-Jin Ra, Sang-Mi Jung, Mi-Jeong Ahn, Seulah Lee, Ki Hyun Kim

**Affiliations:** 1 School of Pharmacy, Sungkyunkwan University, Suwon 16419, Republic of Korea; bbj0423@gmail.com; 2 College of Pharmacy and Research Institute of Pharmaceutical Sciences, Gyeongsang National University, Jinju 52828, Republic of Korea; kdm7105@gnu.ac.kr (D.-M.K.); amj5812@gnu.ac.kr (M.-J.A.); 3 Laboratory of Nuclear Magnetic Resonance, National Center for Inter-University Research Facilities (NCIRF), Seoul National University, Seoul 08826, Republic of Korea; yjko@snu.ac.kr; 4 Hongcheon Institute of Medicinal Herb, Hongcheon 25142, Republic of Korea; ramj90@himh.re.kr (M.-J.R.); sgmo77@naver.com (S.-M.J.); 5 Department of Oriental Medicine Biotechnology, Kyung Hee University, Yongin 17104, Republic of Korea

**Keywords:** *Salix koreensis*, phenolic glycoside, NMR, DP4+ analysis, *Helicobacter pylori*

## Abstract

*Salix koreensis* Anderss (Salicaceae), commonly referred to as Korean willow, is native to East Asia, particularly Korea and China, and it has been used in traditional Korean folk medicine for its potent anti-inflammatory, analgesic, and antioxidant properties. In our ongoing research efforts to discover biologically new natural products, phytochemical analysis on an ethanolic extract of *S. koreensis* twigs yielded the isolation and identification of ten phenolic compounds (**1**–**10**), including a newly discovered phenolic glycoside (**1**) named isograndidentatin D, isolated via HPLC purification. The structure of compound **1** was determined through extensive 1D and 2D NMR spectral data analysis and high-resolution electrospray ionization mass spectrometry (HR-ESIMS). Its absolute configuration was established using DP4+ probability analysis combined with gauge-including atomic orbital NMR chemical shift calculations and chemical reaction methods. The other known compounds were identified as isograndidentatin B (**2**), trichocarposide (**3**), glanduloidin C (**4**), tremuloidin (**5**), 3-*O*-acetylsalicin (**6**), 2-*O*-acetylsalicin (**7**), salicin (**8**), salireposide (**9**), and coumaric acid (**10**), confirmed by comparing their NMR spectra with previously reported data and further verified through liquid chromatography/mass spectrometry (LC/MS) analysis. The isolated compounds **1**–**10** were tested for their anti-*Helicobacter pylori* activities. Among these, compounds **4** and **5** demonstrated moderate anti-*H. pylori* activity at a concentration of 100 μM. Specifically, compound **5** showed an inhibitory activity of 35.9 ± 5.4%, making it slightly more potent than compound **4**, with 34.0 ± 1.0% inhibition. These results were comparable to that of quercetin, a known anti-*H. pylori* agent used as a positive control in this study, which showed 38.4 ± 2.3% inhibition. The remaining compounds exhibited very weak inhibitory effects. This study highlights the potential of *S. koreensis* twigs as a valuable natural source of bioactive compounds for therapeutic applications against *H. pylori*.

## 1. Introduction

*Salix koreensis* Anderss (Salicaceae), commonly known as the Korean willow, is a deciduous broad-leaved tree that thrives in wetlands. It is widely distributed in East Asia, particularly in Korea and China [1]. *S. koreensis* has traditionally been used in Korean folk medicine for its potent anti-inflammatory, analgesic, and antioxidant properties. It was primarily used to treat neuralgia, pain, edema, and inflammatory conditions such as arthritis and fever. The bark and leaves of *S. koreensis* were commonly utilized in decoctions, a method of extracting bioactive compounds by boiling plant materials. These preparations were especially valued for their ability to alleviate pain and reduce inflammation, similarly to other species within the *Salix* genus, which contain salicin, a precursor to salicylic acid, the active ingredient in aspirin [1].

From a natural products chemistry perspective, the exploration of *S. koreensis* is particularly compelling due to its complex secondary metabolite profile. Compared to other *Salix* species, in terms of chemical composition, *S. koreensis* shares many phytochemical similarities but also displays distinctive profiles of secondary metabolites. Common to many species in the *Salix* genus are salicin derivatives, phenolic glycosides, flavonoids, and tannins, which contribute to their medicinal properties, such as their anti-inflammatory and analgesic effects. However, specific studies on *S. koreensis* have revealed unique compounds not found in other *Salix* species, as well as variations in the concentrations of common compounds. For instance, *S. koreensis* exhibits a higher concentration of specific phenolic compounds and flavonoids compared to species like *S. acutifolia* and *S. glandulosa* [2]. Additionally, other *Salix* species such as *S. glandulosa* have been shown to contain novel phenolic compounds like saliglandol, which is less prevalent or absent in *S. koreensis* [3].

The compounds derived from *S. koreensis* and related *Salix* species show a wide range of therapeutic applications, primarily due to their anti-inflammatory, analgesic, and metabolic regulatory properties. One of the primary active compounds found in *Salix* species, including *S. koreensis*, is salicin, a precursor to salicylic acid, which has been used historically to treat pain and inflammation. Recent studies have expanded the therapeutic potential of *Salix* species by identifying specific bioactive compounds, such as 2’-*O*-acetylsalicortin, which exhibits significant anti-inflammatory properties by inhibiting the release of prostaglandin E2 (PGE2), a key mediator in peripheral inflammation [4]. Furthermore, the strong antioxidant properties of phenolic compounds from *Salix* species offer potential for treating oxidative stress-related disorders [5,6], highlighting the broad therapeutic applications of *Salix* species in both traditional and modern medicine.

Modern applications derived from the traditional uses of *S. koreensis* have expanded significantly, especially in the fields of anti-inflammatory therapies, wound healing, and antioxidant treatments. For instance, extracts from *S. koreensis* have been studied for their wound-healing properties, particularly their ability to promote collagen production and keratinocyte proliferation, which are crucial for skin repair [1]. Additionally, *S. koreensis* extracts have shown strong antioxidant and anti-inflammatory effects, making them suitable for inclusion in modern food additives and pharmaceutical formulations targeting oxidative stress and inflammation-related conditions [7]. These bioactive properties align with the traditional use of *Salix* species in treating pain, fever, and inflammatory disorders, now applied in more-precise therapeutic contexts such as anti-aging skincare and dietary supplements. In addition, *S. koreensis* leaf extract has been demonstrated to reduce inflammatory markers and enhance the antioxidant defense system in cellular models, suggesting it can be a potent natural additive to extend shelf life and improve the nutritional profile of food products [7]. This makes *S. koreensis* a promising candidate for inclusion in health-oriented food formulations. 

In addition, previous studies on various *Salix* species have demonstrated promising antibacterial activities. For instance, a methanol extract from *S. terasperma* stem bark exhibited anti-quorum sensing activity against *Pseudomonas aeruginosa* by inhibiting bacterial motility and virulence factors, suggesting its potential role in overcoming antibiotic resistance [8]. Similarly, aqueous extracts of twigs and leaves from *S. babylonica* have shown notable in vitro antimicrobial activity against gram-negative bacteria, including *Escherichia coli* and *Salmonella enterica*, as well as the gram-positive yeast *Candida albicans* [9]. Furthermore, a hydroalcoholic extract of *S. babylonica* and its derived fractions displayed significant antibacterial activity against *E. coli*, *Staphylococcus aureus*, and *Listeria monocytogenes*. Luteolin and luteolin-7-*O*-glucoside were identified as the antibacterial compounds found in *S. babylonica* extracts, highlighting its potential as an alternative treatment option [10].

However, studies focusing exclusively on the bioactive compounds in *S. koreensis* are limited, and insights regarding the unique bioactive compounds identified in other *Salix* species could enhance understanding of the bioactive potential of *S. koreensis* [11]. Although comprehensive phytochemical studies on *S. koreensis* are scarce, the species likely harbors unique phenolic compounds, flavonoids, and their glycosides that contribute to its medicinal properties. Thus, there is a significant need to identify new bioactive compounds with diverse skeletal structures from *S. koreensis*.

In our ongoing quest to discover novel and bioactive metabolites from natural sources [12,13,14,15,16], we have initiated a detailed investigation into an ethanol (EtOH) extract of *S. koreensis* twigs. This study aimed to isolate and identify bioactive phenolic compounds from the EtOH extract through comprehensive phytochemical analysis using column chromatography and semi-preparative high-performance liquid chromatography (HPLC). Furthermore, the study was extended to evaluate the anti-*Helicobacter pylori* activities of the isolated compounds. Herein, we describe the isolation and structural elucidation of compounds **1**–**10** and the assessment of their antibacterial activities, providing insights into their potential therapeutic applications.

## 2. Results and Discussion

### 2.1. Isolation and Structural Elucidation of Compounds ***1**–**10***

**Figure 1:**
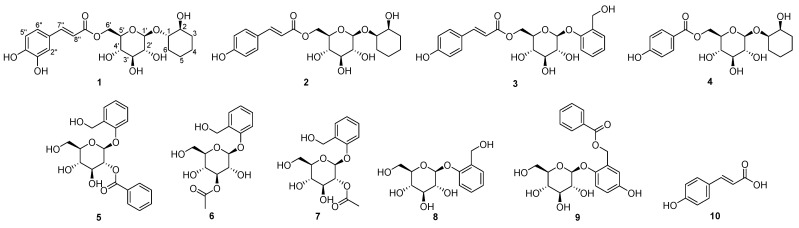
Chemical structures of compounds **1**–**10**.

The twigs of *S. koreensis* were extracted with 80% ethanol, producing an ethanol (EtOH) extract that was subsequently fractionated by solvent partitioning into *n*-hexane-, dichloromethane (CH_2_Cl_2_)-, ethyl acetate (EtOAc)-, and *n*-butanol (*n*-BuOH)-soluble fractions. The EtOAc fraction was selected for detailed study due to its rich content of phenolic glycosides, as identified through LC/MS and HR-LC/MS analyses. This fraction underwent extensive phytochemical investigation, involving repeated column chromatography and both preparative and semi-preparative HPLC. These purification steps resulted in the isolation of a new phenolic glycoside (**1**) along with nine known phenolic compounds (**2**–**10**), as depicted in Figure 1.

Compound **1** was isolated as a colorless gum, with its molecular formula determined to be C_21_H_28_O_10_, based on the molecular ion peak at [M + H]^+^ *m*/*z* 441.1767 (calcd. for C_21_H_29_O_10_, 441.1755), attained using positive-ion high-resolution electrospray ionization mass spectrometry. The ^1^H NMR data collected from **1** (Table 1) showed the presence of a 1,3,4-trisubstituted aromatic ring [*δ*_H_ 7.05 (1H, d, *J* = 2.0 Hz), 6.95 (1H, dd, *J* = 8.0, 2.0 Hz) and 6.78 (1H, d, *J* = 8.0 Hz)], a *trans*-olefinic bond [*δ*_H_ 7.61 (1H, d, *J* = 16.0 Hz) and 6.30 (1H, d, *J* = 16.0 Hz)], an anomeric proton [*δ*_H_ 4.42 (1H, d, *J* = 8.0)], two deshielded oxygenated methines [*δ*_H_ 4.59 (1H, dd, *J* = 12.0, 2.0 Hz) and 4.24 (1H, dd, *J* = 12.0, 6.5 Hz)], oxygenated methines attributable to the sugar moiety between *δ*_H_ 3.24 and 3.58 ppm, and alkyl protons between *δ*_H_ 1.25 and 2.06 ppm. The ^13^C NMR data (Table 1) obtained with the assistance of HSQC and HMBC spectra further confirmed the presence of a carbonyl carbon (*δ*_C_ 169.0), a 1,3,4-trisubstituted aromatic ring (*δ*_C_ 149.7, 146.9, 127.8, 123.1, 116.5, and 115.1), a *trans*-olefinic bond (*δ*_C_ 147.3 and 114.8), a sugar moiety (*δ*_C_ 103.4, 77.7, 75.6, 74.7, 71.7, and 64.7), and a 1,2-cyclohexanediol (*δ*_C_ 85.7, 74.3, 33.4, 31.5, 25.1, and 24.8). Based on this information, compound **1** was predicted to be a phenolic glycoside that is structurally similar to isograndidentatin B (**2**) [17], with an extra hydroxyl group substituted to the aromatic ring.

**Figure 2:**
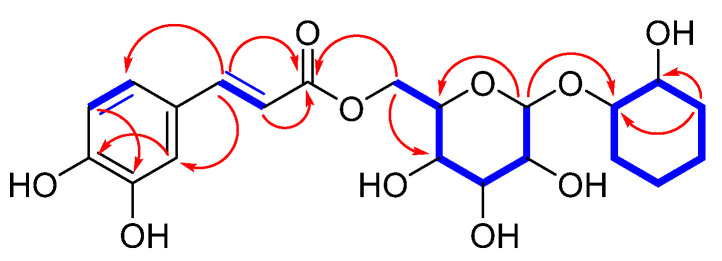
Key ^1^H-^1^H COSY (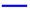) and HMBC (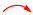) correlations for compound **1**.

The planar structure of compound **1** was elucidated through 2D NMR spectroscopy analysis, specifically utilizing COSY and HMBC experiments. These techniques provided detailed insights into the connectivity and arrangement of the atoms within the molecule, enabling the construction of the molecular framework illustrated in Figure 2. The HMBC correlations between H-2″/C-4″, H-5″/C-3″, and H-7″/C-2″ and C-6″ confirmed the presence of a 1,3,4-trisubstituted aromatic ring. Further analysis of the HMBC and ^1^H-^1^H COSY spectra revealed that the complete planar structure of compound **1** shares the same sugar moiety connectivity (glucose) and 1,2-cyclohexanediol unit as those found in isograndidentatin B (**2**). These structural similarities were detailed and corroborated using the spectral data, as illustrated in Figure 2. To confirm the sugar unit, *β*-glucosidase (1 mg, almonds, Sigma-Aldrich) was utilized for the enzymatic hydrolysis of **1**. The subsequent enzymatic hydrolysis gave D-glucopyranose, which was confirmed by LC/MS analysis [18,19]. Finally, the coupling constant of the anomeric proton at *δ*_H_ 4.42 (*J* = 8.0) confirmed the sugar to be *β*-D-glucopyranose.

**Figure 3:**
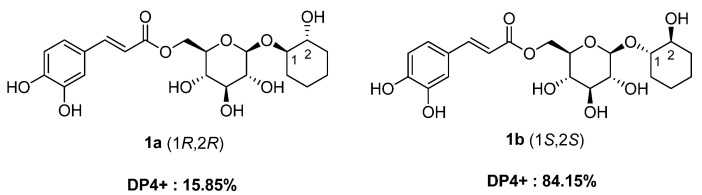
DP4+ analysis and probability scores for compound **1** with **1a**/**1b**.

Careful analysis of the ^13^C NMR data for compound **1** indicated that the chemical shifts of C-1 and C-2 were downfield shifted compared to those of *trans*-glanduloidin F (*δ*_C_ 85.7 and 74.3 for **1** and *δ*_C_ 81.7 and 70.6 for *trans*-glanduloidin F) [20,21,22]. The downfield shifts in compound **1** are characteristic of a *trans*-1,2-cyclohexanediol configuration. This configuration differs notably from the *cis* forms of similar structures, which typically exhibit upfield shifts, as demonstrated by the chemical shifts recorded for compound **2**. To further confirm the absolute configurations of C-1 and C-2 in compound **1**, DP4+ analysis was employed utilizing gauge-including atomic orbital (GIAO) NMR chemical shift calculations. The computationally calculated ^1^H and ^13^C NMR chemical shifts for the two possible diastereomers, **1a** (1*R*, 2*R*) and **1b** (1*S*, 2*S*), were compared with the experimental data for compound **1**. The DP4+ probability analysis strongly supported the structural equivalence of compound **1** to diastereomer **1b** (1*S*, 2*S*) with a probability of 84.15%, as depicted in Figure 3. Thus, based on these comprehensive data, the chemical structure of compound **1** was confidently determined; it is illustrated in Figure 1 and named isograndidentatin D.

In our investigation of *S. koreensis* twigs, we successfully identified a series of known phenolic compounds based on their NMR spectra and liquid chromatography/mass spectrometry (LC/MS) analysis. The identified compounds are as follows: isograndidentatin B (**2**) [17], trichcarposide (**3**) [23], glanduloidin C (**4**) [20], tremuoidin (**5**) [24], 3-*O*-acetylsalicin (**6**) [25], 2-*O*-acetylsalicin (**7**) [26], salicin (**8**) [27], salireposide (**9**) [28] and cumaric acid (**10**) [29].

### 2.2. Evaluation of Anti-Helicobacter pylori Activity of the Isolated Compounds

Historically, extracts from various *Salix* species have shown antimicrobial efficacy against both gram-positive bacteria, such as *Staphylococcus aureus* and *Bacillus subtilis*, and gram-negative bacteria, including *Escherichia coli*, *Klebsiella pneumoniae*, *Salmonella enterica*, and *Pseudomonas aeruginosa* [6,30,31]. Despite the known potential of *Salix* species, there has been no research specifically targeting the anti-*Helicobacter pylori* compounds found in *S. koreensis*. *H. pylori* is a prevalent pathogen linked to gastritis, duodenal ulcers, and gastric cancer [32,33,34]. Current treatments typically involve triple therapy, combining a proton pump inhibitor with two antibiotics, such as amoxicillin and clarithromycin or metronidazole, but these treatments can lead to side effects including antibiotic resistance and gastrointestinal issues [35]. Given the rising concerns over adverse effects associated with conventional treatments, there is increasing demand for natural products as adjuvant therapies. According to existing research, phenolic glycosides have shown antimicrobial activity against bacteria such as *Staphylococcus aureus*, *Enterococcus faecalis*, and *Bacillus cereus* [36,37]. In addition, a recent study by our group demonstrated that phenolic glycosides isolated from the leaves of *S. chaenomeloides*, including chaenomelin, arbutin cinnamate, and 1-[*O*-β-D-glucopyranosyl-(1 → 2)-β-D-glucopyranosyl]oxy-2-phenol, exhibited antibacterial activity against *H. pylori* [38]. Furthermore, phenolic glycosides from other plant sources have also been reported to possess anti-*H. pylori* activity [39,40]. Based on these findings, this study evaluated antimicrobial activity against *H. pylori* strain 51 for the isolated compounds (**1**–**10**), the majority of which were phenolic glycosides from *S. koreensis* twigs. With this approach, we aimed to broaden our understanding of the antimicrobial scope of phenolic glycosides and to potentially identify new therapeutic options for treating infections caused by *H. pylori*. As benchmarks, metronidazole, a clinically used antibiotic, and quercetin, a natural agent with known anti-*H. pylori* effects, were used as positive controls [41,42]. Among the tested compounds, compounds **4** and **5**, identified as glanduloidin C (**4**) and tremuloidin (**5**), respectively, displayed moderate anti-*H. pylori* activity at a 100 μM concentration (Table 2). Notably, the inhibition by tremuloidin (35.9 ± 5.4%) was more potent than that of glanduloidin C (34.0 ± 1.0%), and both were comparable to quercetin (38.4 ± 2.3%). The other compounds showed much weaker inhibition, ranging from 2.9% to 28.7%. This study marks the first report of anti-*H. pylori* activity for glanduloidin C and tremuloidin, highlighting the potential of natural products as adjuvant therapies with reduced adverse effects.

## 3. Materials and Methods

### 3.1. Equipment Used for Analyses

The equipment and devices used in the analyses and experiments are listed in Table S1.

### 3.2. Plant Material

The twigs of *S. koreensis* were collected in May 2021 from Goesan-gun, Chungcheongbuk-do, Republic of Korea. A voucher specimen, labeled HIMH-2108, was authenticated by Dr. Hye-Ryen Na at the Northeastern Asia Biodiversity Institute, located in Seoul 05677, Republic of Korea. The collected material has been preserved and is available at the herbarium of the Nakdonggang National Institute of Biological Resources in Sangju, Republic of Korea.

### 3.3. Extraction and Isolation

The dried twigs of *S. koreensis* (1.5 kg) were extracted with 80% ethanol (10 L) under reflux at 65 °C for 2 h. The extract was concentrated using a rotary evaporator, yielding a brown crude extract (92.4 g, extraction yield 6.2%). This crude extract (92.4 g) was dissolved in distilled water (700 mL) and subsequently partitioned with *n*-hexane, dichloromethane (CH_2_Cl_2_), ethyl acetate (EtOAc), and *n*-butanol (*n*-BuOH), resulting in four layers of increasing polarity [43,44,45,46]. The yields were 19.0 g (yield 20.6%) for *n*-hexane-, 5.2 g (yield 5.6%) for CH_2_Cl_2_-, 5.1 g (yield 5.5%) for EtOAc-, and 10.0 g (yield 10.8%) for *n*-BuOH-soluble fractions. It was confirmed via analysis of LC/MS and HR-LC/MS data from the fractions that the EtOAc-soluble fraction (5.1 g) predominantly contains phenolic glycosides. 

The EtOAc fraction underwent normal-phase silica open-column chromatography using a gradient solvent system from CH_2_Cl_2_/MeOH (20:1) to 100% MeOH, resulting in six fractions (1–6) [Fraction 1: CH₂Cl₂/MeOH (20:1), Fraction 2: CH₂Cl₂/MeOH (10:1), Fraction 3: CH₂Cl₂/MeOH (5:1), Fraction 4: CH₂Cl₂/MeOH (3:1), Fraction 5: CH₂Cl₂/MeOH (1:1), and Fraction 6: 100% MeOH]. Fraction 3 (3.2 g) was further separated by reversed-phase silica open-column chromatography using a MeOH/H_2_O gradient (40% to 100% MeOH), yielding six subfractions (3.1–3.6). Subfraction 3.5 (677 mg) was subjected to normal-phase silica open-column chromatography with a CH_2_Cl_2_/MeOH gradient (40:1 to 100% MeOH), producing five subfractions (3.5.1–3.5.5). Subfraction 3.5.1 (303 mg) was further purified using Sephadex LH-20 chromatography with 100% MeOH, leading to four subfractions (3.5.1.1–3.5.1.4). Subfraction 3.5.1.1 (170 mg) underwent another round of normal-phase silica open-column chromatography with a CH_2_Cl_2_/MeOH gradient (20:1 to 100% MeOH), resulting in four subfractions (3.5.1.1.1–3.5.1.1.4). Finally, subfraction 3.5.1.1.1 (54 mg) was processed through semi-preparative reversed-phase HPLC with a 30% MeOH/H_2_O isocratic system to isolate compounds **6** (2.3 mg, *t*_R_ = 21.2 min, 0.000153%) and **7** (2.7 mg, *t*_R_ = 37.3 min, 0.000180%). Subfraction 3.5.1.1.2 (23 mg) underwent semi-preparative reversed-phase HPLC with a 40% MeOH/H_2_O isocratic solvent system, resulting in the isolation of compound **4** (0.4 mg, *t*_R_ = 50.5 min, 0.000026%). Subfraction 3.5.1.1.3 (35 mg) was processed using semi-preparative reversed-phase HPLC with a 15% MeOH/H_2_O isocratic system to isolate compound **8** (4.0 mg, *t*_R_ = 37.4 min, 0.000266%). Subfraction 3.5.2 (240 mg) was subjected to reversed-phase silica open-column chromatography using a MeOH/H_2_O gradient (10–100% MeOH), yielding three fractions (3.5.2.1–3.5.2.3). Subfraction 3.5.2.2 (119 mg) underwent preparative reversed-phase HPLC with a MeOH/H_2_O gradient (45% to 60% MeOH), leading to three fractions (3.5.2.2.1–3.5.2.2.3). Subfraction 3.5.2.2.2 (28 mg) was processed using semi-preparative reversed-phase HPLC with a 16% MeCN/H_2_O isocratic system to isolate compound **10** (1.3 mg, *t*_R_ = 40.3 min, 0.000086%). Subfraction 3.6 (528 mg) was subjected to Sephadex LH-20 open-column chromatography with a 100% MeOH isocratic system, producing seven fractions (3.6.1–3.6.7). Subfraction 3.6.3 (158 mg) underwent semi-preparative reversed-phase HPLC with a 22% MeCN/H_2_O isocratic system, resulting in the isolation of compounds **1** (2.0 mg, *t*_R_ = 21.2 min, 0.000133%), **2** (1.0 mg, *t*_R_ = 35.5 min, 0.000066%), **3** (0.5 mg, *t*_R_ = 53.8 min, 0.000033%), **5** (0.4 mg, *t*_R_ = 56.7 min, 0.000026%), and **9** (0.5 mg, *t*_R_ = 32.5 min, 0.000033%).

#### Isograndidentatin D (**1**)

Colorless gum; [${\alpha ]}_{D}^{25}$-74 (*c* 0.08, MeOH); UV (MeOH): λ_max_ (log *ε*) = 326 (3.7), 219 (2.6) nm; IR (neat): *ν*_max_ = 3391, 2939, 1698, 1603, 1157, 1081, 992 cm^−1^; ^1^H (850 MHz) and ^13^C (212.5 MHz) NMR data, see Table 1; HR-ESIMS (positive-ion mode) *m*/*z* 441.1767 [M + H]^+^ (calcd. for C_21_H_29_O_10_, 441.1755).

### 3.4. Enzymatic Hydrolysis and Sugar Identification of ***1***

To determine the absolute configuration of the sugar moiety in compound **1**, 1.0 mg of the compound was hydrolyzed using β-glucosidase (10 mg, Sigma-Aldrich, St. Louis, MO, USA) from almonds, dissolved in distilled water [18,19]. The hydrolysis reaction was carried out at 37 °C for 72 h in a dry oven. After cooling, the aglycone was extracted via solvent partitioning with ethyl acetate (EtOAc). The aqueous layer was evaporated under vacuum, redissolved in 0.5 mL of anhydrous pyridine, and reacted with 1 mg of L-cysteine methyl ester chloride. The mixture was heated at 60 °C for 1 h, followed by the addition of 50 μL of *O*-tolylisothiocyanate, and heated for an additional hour at the same temperature. The reaction mixture was then concentrated, redissolved in methanol, and analyzed by LC/MS using an analytical Kinetex C18 100 Å column (100 mm × 2.1 mm i.d., 5 μm). A gradient solvent system of 0% to 80% MeOH/H₂O over 30 min was employed for the analysis. For comparison, standard substances of D-glucose and L-glucose underwent the same derivatization process and were analyzed under identical conditions. Based on the retention times in the LC/MS analysis, the sugar moiety in compound **1** was identified as D-glucose, with compound **1** showing a retention time of 21.4 min, compared to 21.3 min for D-glucose and 20.7 min for L-glucose.

### 3.5. Computational NMR Chemical Shift Calculations for DP4+ Analysis

The DP4+ probability analysis was conducted on the geometrically optimized conformers of diastereomers **1a** and **1b** using density functional theory (DFT). The detailed procedure is included in the Supplementary Materials.

### 3.6. Anti-Helicobacter pylori Activity

The *H. pylori* strain 51 isolated from a Korean patient diagnosed with a duodenal ulcer (HPKTCC B0006) was obtained from the Korean Type Culture Collection at the School of Medicine, Gyeongsang National University, Korea. This clinical isolate was propagated and maintained in Brucella broth medium (BD Co., Sparks, MD, USA) supplemented with 10% horse serum (Gibco, New York, NY, USA). The cultures were incubated at 37 °C in an atmosphere of 100% relative humidity and 10% CO_2_. The assessment of antibacterial activity was conducted following a previously established methodology [47,48]. Briefly, each well of a six-well plate was inoculated with 20 μL of a bacterial suspension, adjusted to 2–3 × 10^8^ cfu/mL, and placed in Brucella broth medium enriched with 10% horse serum. The samples and positive controls were administered at a final concentration of 100 μM. The total volume in each well was maintained at 2 mL. The plate was incubated under the same conditions used for bacterial culture (37 °C, 100% humidity and 10% CO_2_) for 24 h. Bacterial proliferation was evaluated by measuring optical density at 600 nm using an Optizen POP UV/VIS spectrophotometer (Mecasys, Daejeon, Korea). The anti-*H. pylori* activity was quantified using the formula:

Inhibition (%) = [(absorbance of the negative control − absorbance of solution with samples)/absorbance of the negative control] × 100

Dimethyl sulfoxide (DMSO) served as the negative control, while quercetin and metronidazole (Sigma, St. Louis, MO, USA) were employed as positive controls. The final concentration of DMSO was 1%, which did not show any effect on the growth of *H. pylori* [49]. Data were expressed as means ± standard deviations (SD) of the double-independent experiments. The statistical differences among the samples were determined by Tukey’s range test using IBM SPSS Statistics 24.0 software (Armonk, New York, NY, USA). The statistical significance level used was 5%.

## 4. Conclusions

In this study, we explored the bioactive constituents of *S. koreensis* twigs, leading to the discovery of a novel phenolic glycoside, designated isograndidentatin D (**1**), and the identification of other known compounds: isograndidentatin B (**2**), trichocarposide (**3**), glanduloidin C (**4**), tremuloidin (**5**), 3-*O*-acetylsalicin (**6**), 2-*O*-acetylsalicin (**7**), salicin (**8**), salireposide (**9**), and coumaric acid (**10**). Upon verification, all the isolated compounds, except for compound **2**, were reported for the first time from *S. koreensis*. The structural elucidation of the new compound was achieved through comprehensive 1D and 2D NMR spectroscopy, high-resolution electrospray ionization mass spectrometry (HR-ESIMS), DP4+ probability analysis, and various chemical reactions. The antimicrobial efficacy of these isolated compounds against *H. pylori* was subsequently assessed. Notably, compounds **4** and **5** exhibited moderate anti-*H. pylori* activity, comparable to that of quercetin, which served as a positive control. While quercetin is widely available, the unique structural features of compounds **4** and **5** may provide distinct mechanisms of action or synergistic effects when combined with other treatments for *H. pylori* therapy. These findings serve as foundational data for future studies aimed at developing natural product-based anti-*H. pylori* agents with novel chemical scaffolds and enhanced potency. Furthermore, the observed biological activities of these isolates provide valuable insights into the potential therapeutic applications of *S. koreensis* twigs, emphasizing their promise as antimicrobial agents with specific efficacy against *H. pylori*.

## Figures and Tables

**Table 1:** ^1^H (850 MHz) and ^13^C NMR (212.5 MHz) data for compound **1** in CD_3_OD (*δ* ppm) ^a^.

Position	1	
	*δ*_H_ (*J* in Hz)	*δ*_C_
1	3.40, overlap	85.7 CH
2	3.41, overlap	74.3 CH
3	1.98, m; 1.25, overlap	33.4 CH_2_
4	1.66, overlap; 1.25, overlap	24.8 CH_2_
5	1.70, overlap; 1.24, overlap	25.1 CH_2_
6	2.06, m; 1.33, overlap	31.5 CH_2_
1′	4.42, d (8.0)	103.4 CH
2′	3.24, dd, (9.0, 8.0)	74.7 CH
3′	3.39, overlap	77.7 CH
4′	3.35, overlap	71.7 CH
5′	3.58, m	75.6 CH
6′	4.59, dd (12.0, 2.0); 4.24, dd (12.0, 6.5)	64.7 CH_2_
1″		127.8 C
2″	7.05, d (2.0)	115.1 CH
3″		146.9 C
4″		149.7 C
5″	6.78, d (8.0)	116.5 CH
6″	6.95, dd (8.0, 2.0)	123.1 CH
7″	7.61, d (16.0)	147.3 CH
8″	6.30, d (16.0)	114.8 CH
9″		169.0 C

^a^ Coupling constants (Hz) are given in parentheses and ^13^C NMR data are assigned based on HSQC and HMBC experiments.

**Table 2:** Antimicrobial activities of compounds **1–10** against *H. pylori* strain 51 treated with 100 μM of each compound.

Compound	Inhibition (%) ^B^
**1**	20.6 ± 2.5 ^d^
**2**	28.7 ± 0.2 ^c^
**3**	17.9 ± 0.5 ^d^
**4**	34.0 ± 1.0 ^b^
**5**	35.9 ± 5.4 ^b^
**6**	8.1 ± 4.9 ^e^
**7**	20.6 ± 0.0 ^d^
**8**	7.1 ± 1.0 ^e^
**9**	2.9 ± 3.2 ^e^
**10**	16.4 ± 6.0 ^d^
**Quercetin** **^A^**	38.4 ± 2.3 ^b^
**Metronidazole** **^A^**	96.6 ± 0.5 ^a^

**^A^** Positive controls; **^B^** different letters in the same column indicate significant difference (*p* < 0.05).

## Data Availability

The original contributions presented in this study are included in the article/supplementary material. Further inquiries can be directed to the corresponding authors.

## Supplementary Materials

The following are available online at https://www.mdpi.com/article/10.3390/plants13243603/s1: Figure S1: The HR-ESIMS data of compound **1**; Figure S2: The UV spectrum of compound **1**; Figure S3. The ^1^H NMR spectrum of compound **1** (CD_3_OD, 850 MHz); Figure S4. The ^13^C NMR spectrum of compound **1** (CD_3_OD, 212.5 MHz); Figure S5: The ^1^H-^1^H COSY spectrum of compound **1**; Figure S6: The HSQC spectrum of compound **1**; Figure S7: The HMBC spectrum of compound **1**; Table S1: Equipment used for analyses and computational NMR chemical shift calculations for DP4+ analysis. References [50,51] are cited in the supplementary materials.
