# Supplementary material for: Discovery of Isograndidentatin D, a Novel Phenolic Glycoside, and Anti-Helicobacter pylori Phenolics from Salix koreensis Twigs
Source: Plants (Basel). 2024 Dec 23;13(24):3603. doi: 10.3390/plants13243603 (PMC11678160; doi:10.3390/plants13243603)
Supplement: Supplementary file 1 [file plants-13-03603-s001.zip › plants-3372035-supplementary.pdf]

Supplementary data

Discovery of Isograndidentatin D, a Novel Phenolic Glycoside, and Anti-*Helicobacter pylori* Phenolics from *Salix koreensis* Twigs

Yoon Seo Jang [1], Dong-Min Kang [2], Yoon-Joo Ko [3], Moon-Jin Ra [4], Sang-Mi Jung [4], Mi-Jeong Ahn [2], Seulah Lee [5,*] , Ki Hyun Kim [1,*]

[1] School of Pharmacy, Sungkyunkwan University, Suwon 16419, Republic of Korea; bbj0423@gmail.com (Y.S.J.).
[2] College of Pharmacy and Research Institute of Pharmaceutical Sciences, Gyeongsang National University, Jinju 52828, Republic of Korea; kdm7105@gnu.ac.kr (D.M.K.); amj5812@gnu.ac.kr (M.J.A.).
[3] Laboratory of Nuclear Magnetic Resonance, National Center for Inter-University Research Facilities (NCIRF), Seoul National University, Gwanak-gu, Seoul 08826, Republic of Korea; yjko@snu.ac.kr (Y.J.K.)
[4] Hongcheon Institute of Medicinal Herb, Hongcheon-gun, Gangwon-do 25142, Republic of Korea; ramj90@himh.re.kr (M.J.R.); sgmo77@naver.com (S.M.J.)
[5] Department of Oriental Medicine Biotechnology, Kyung Hee University, Yongin 17104, Republic of Korea
* Correspondence: khkim83@skku.edu (K.H.K.); +82-31-290-7700 (K.H.K.); lee.seulah@khu.ac.kr (S.L.).

Electronic Supplementary Information Contents:

Figure S1. The HR-ESIMS data of compound **1**............S3
Figure S2. The UV spectrum of compound **1**............S4
Figure S3. The ^{1}H NMR spectrum of compound **1** (CD_3OD, 850 MHz) S5
Figure S4. The ^{13}C NMR spectrum of compound **1** (CD_3OD, 212.5 MHz) S6
Figure S5. The ^{1}H-^{1}H COSY spectrum of compound **1**............S7
Figure S6. The HSQC spectrum of compound **1**............S8
Figure S7. The HMBC spectrum of compound **1**............S9
Table S1. Equipment used for analyses S10
Computational NMR chemical shift calculations for DP4+ analysis S11

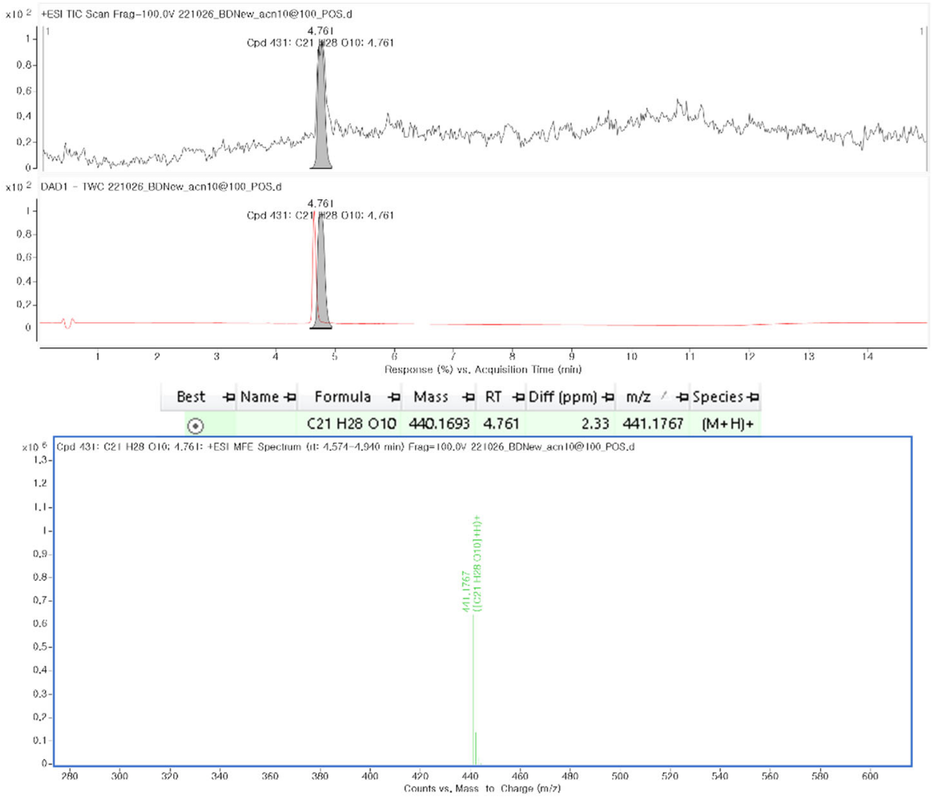

Figure S1. The HR-ESIMS data of compound **1**.

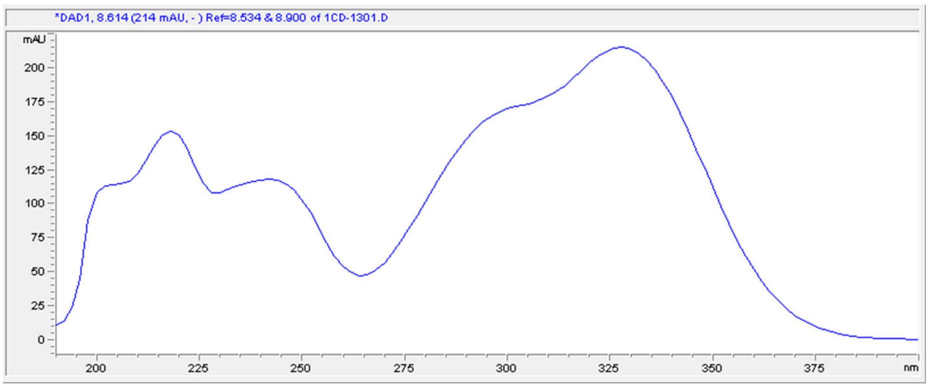

Figure S2. The UV spectrum of compound **1**.

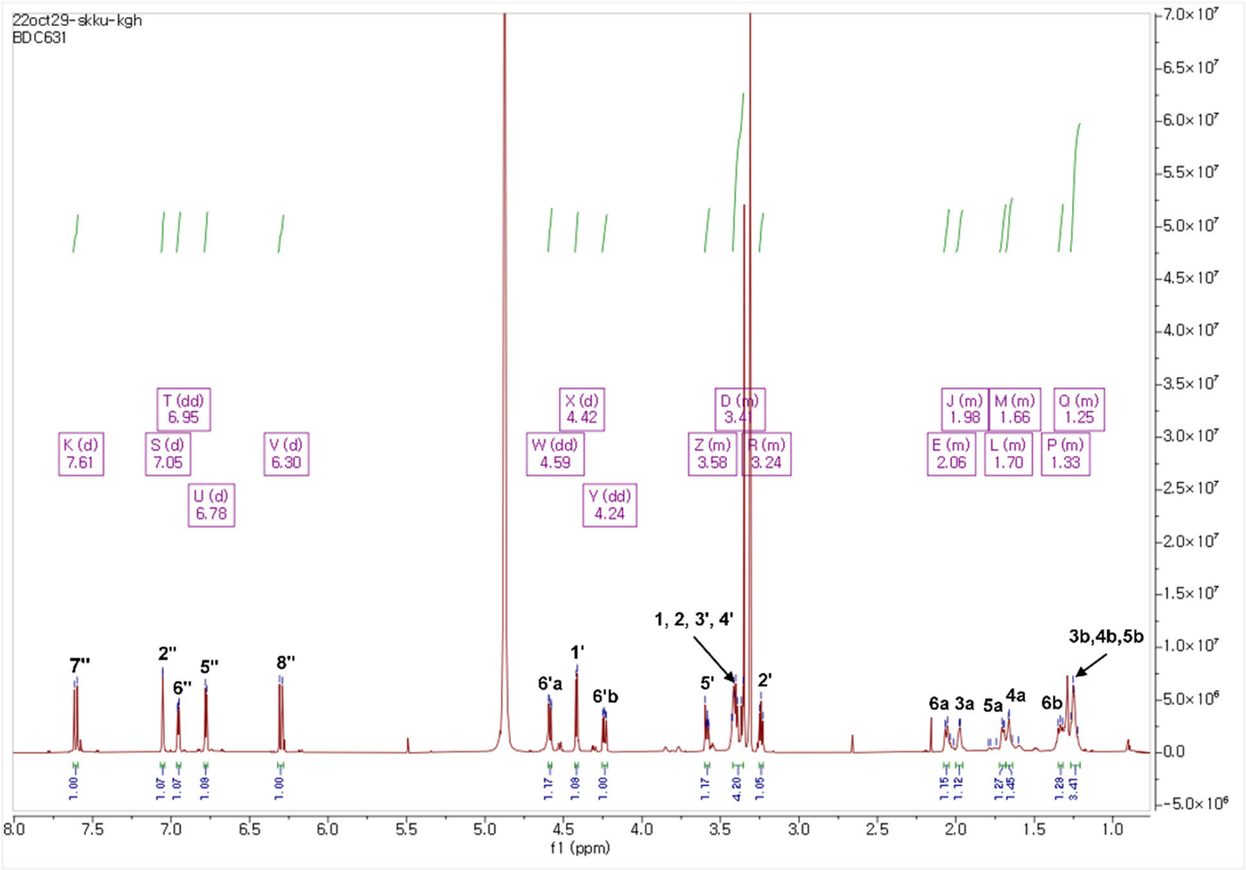

Figure S3. The ^{1}H NMR spectrum of compound **1** (CD_3OD, 850 MHz).

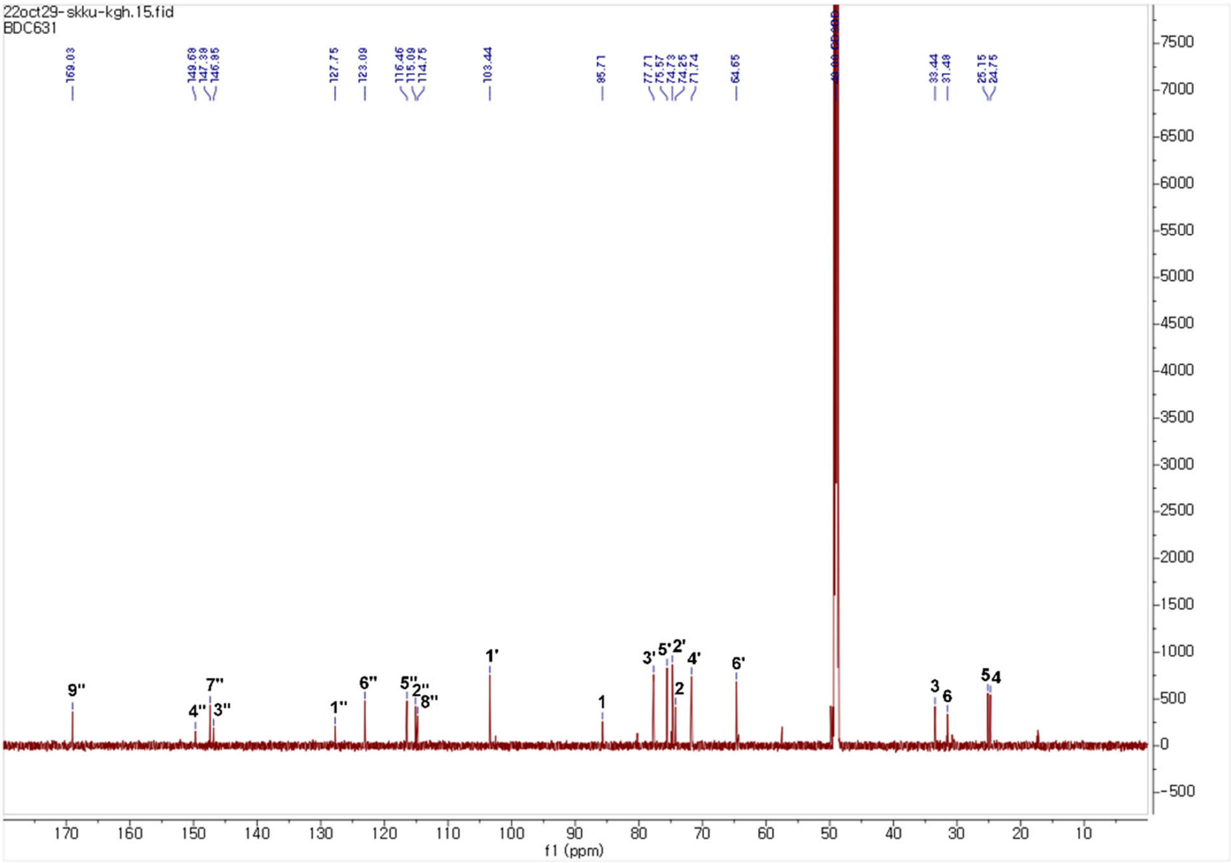

Figure S4. The ^{13}C NMR spectrum of compound **1** (CD_3OD, 212.5 MHz).

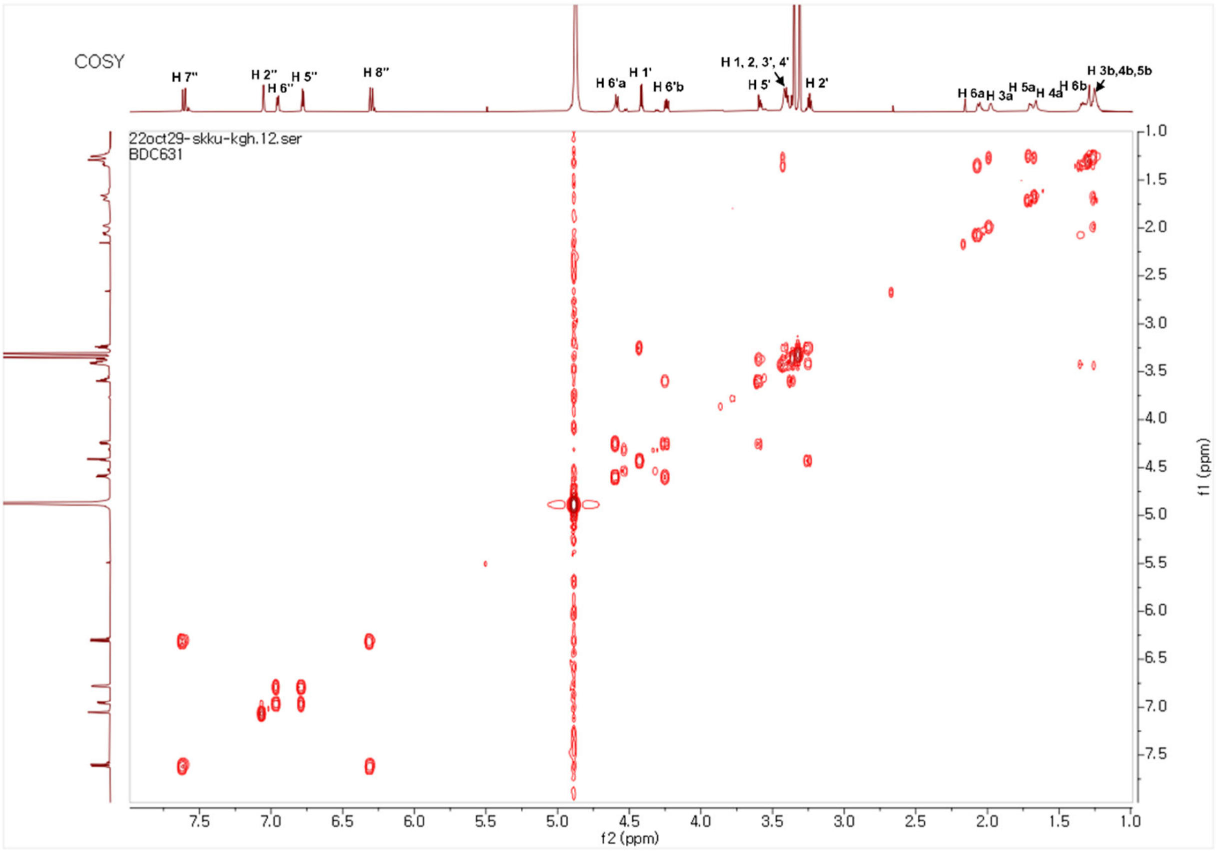

Figure S5. The ^{1}H-^{1}H COSY spectrum of compound **1**.

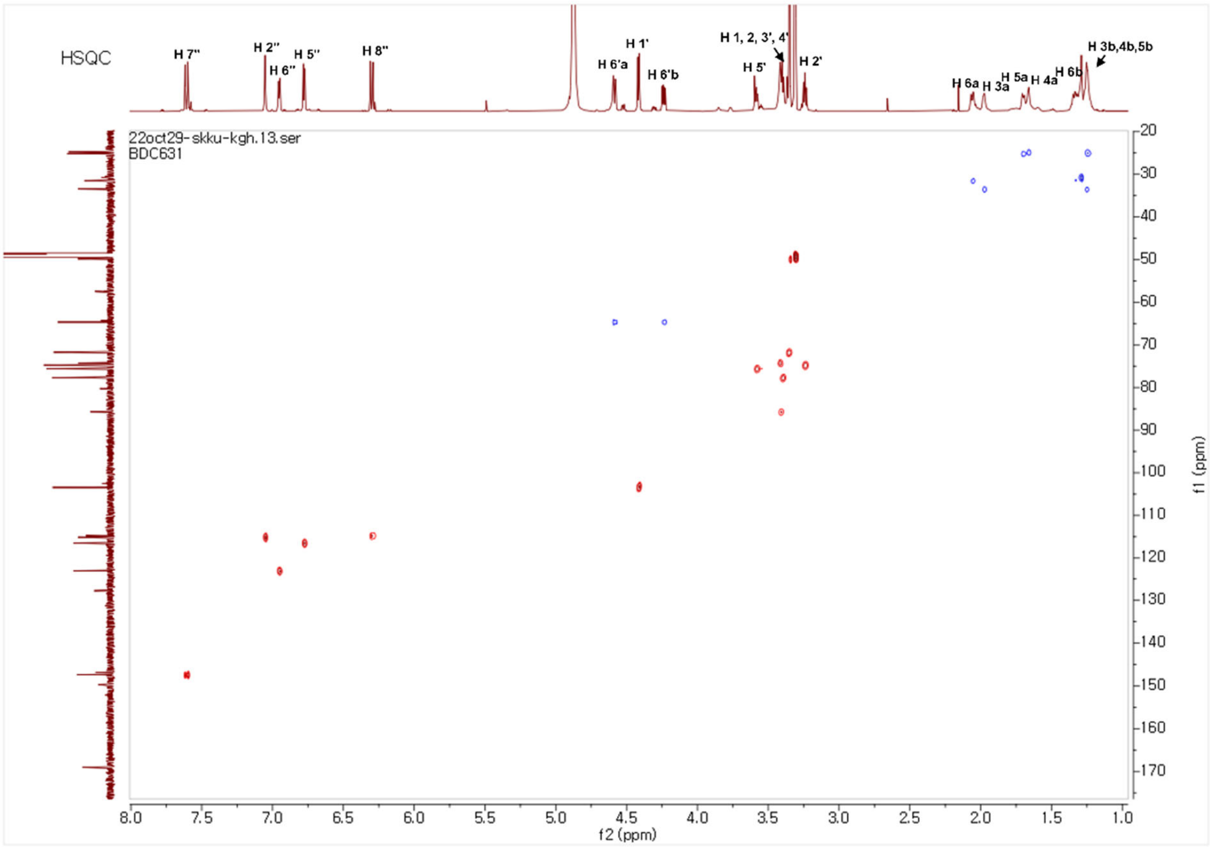

Figure S6. The HSQC spectrum of compound **1**.

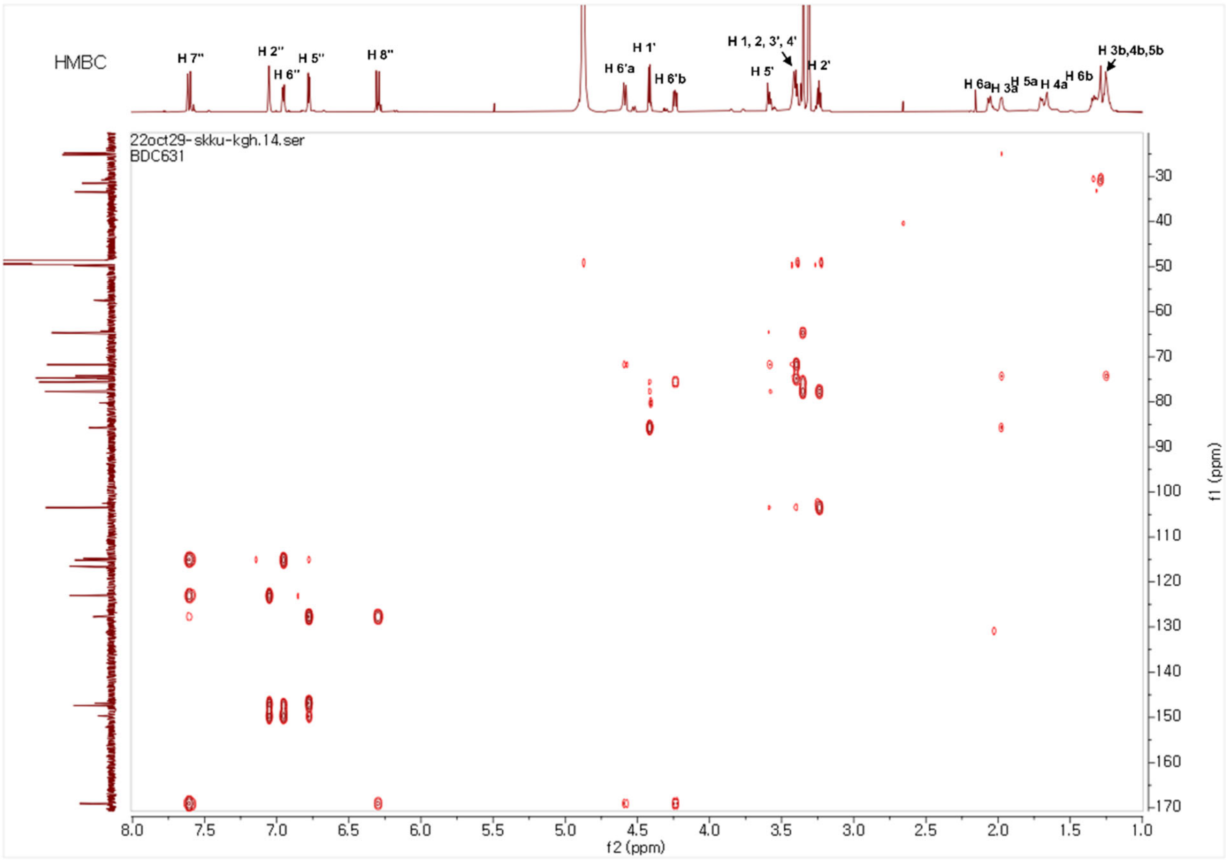

Figure S7. The HMBC spectrum of compound **1**.

Table S1. Equipment used for analyses

**Experimental procedure**	**Equipment**
Optical rotations	JASCO P-2000 polarimeter (JASCO, Easton, MD, USA)
Ultraviolet (UV) spectra	Agilent 8453 UV-visible spectrophotometer (Agilent Technologies, Santa Clara, CA, USA)
Infrared (IR) spectra	Bruker IFS-66/S FT-IR spectrometer (Bruker, Karlsruhe, Germany)
Nuclear magnetic resonance (NMR) spectra	Bruker AVANCE III HD 850 NMR spectrometer with a 5 mm TCI CryoProbe operating at 850 MHz (^{1}H) and 212.5 MHz (^{13}C)
HR-ESIMS	•Agilent G6545B quadrupole time-of-flight mass spectrometer (Agilent Technologies)<br>•Agilent 1290 Infinity II high-performance liquid chromatography (HPLC) instrument (Agilent Eclipse Plus C18 column (2.1 × 50 mm, 1.8 μm; flow rate: 0.3 mL/min))
Preparative HPLC	Waters 1525 Binary HPLC pump with a Waters 996 Photodiode Array Detector (Waters Corporation, Milford, MA, USA) and a Hector C18 column (250 × 21.2 mm, 5 μm; flow rate: 5 mL/min; Rstech Corporation, Korea)
Semi-preparative HPLC	Waters 1525 Binary HPLC pump with a Waters 996 Photodiode Array Detector (Waters Corporation, Milford, CT, USA)<br>•Phenomenex Luna C18 column (250 × 10 mm, 10 μm; flow rate: 2 mL/min;

	Phenomenex, Torrance, CA, USA)<br>•Phenomenex Luna Phenyl-Hexyl column (250 × 10 mm, 10 µm; flow rate: 2 mL/min; Phenomenex)
LC/MS analysis	Agilent 1200 Series HPLC system equipped with a diode array detector and 6130 Series ESI mass spectrometer using an analytical Kinetex C18 100 Å column (100 × 2.1 mm, 5 µm; flow rate: 0.3 mL/min; Phenomenex).
Column chromatography	•Silica gel 60 (230-40 mesh; Merck, Darmstadt, Germany)<br>•RP-C18 silica gel (Merck, 230-240 mesh)<br>•Sephadex LH-20 (Pharmacia, Uppsala, Sweden)<br>•Diaion HP20 (Mitsubishi Chemical, Tokyo, Japan)
Thin-layer chromatography (TLC)	pre-coated silica gel F254 plates and RP-C18 F254s plates (Merck); spots were detected under UV light or by heating following spraying with anisaldehyde-sulfuric acid.

Computational NMR chemical shift calculations for DP4+ analysis

The DP4+ probability analysis was conducted on the geometrically optimized conformers of diastereomers **1a** and **1b**, utilizing Density Functional Theory (DFT). The calculations were performed using the B3-LYP/6-31+G(d,p) level, as specified in previous studies [1]. An Excel sheet (DP4+) developed by Grimblat et al. [2] was used for calculating the Gauge-Including Atomic Orbital (GIAO) magnetic shielding tensors.

The chemical shift values δ_{calc}^{x} were derived from these magnetic shielding tensors according to the equation below, where σ^{o} is the reference shielding tensor for the proton and carbon nuclei in tetramethylsilane (TMS), and σ^{x} is the calculated shielding tensor for nucleus x:

$$\delta_{calc}^{x} = \sigma^{o} - \sigma^{x}$$

The B3-LYP/6-31+G(d,p) basis set was applied to calculate the shielding tensors. The unscaled NMR properties of the optimized structures were averaged, and the scaled chemical shift values were then calculated using the following formula, where the intercept and slope are empirical scaling factors derived from a regression analysis of calculated vs. experimental shifts:

$$\delta_{scaled} = \frac{\delta_{unscaled} - intercept}{slope}$$

This methodology ensures high precision in aligning the theoretical chemical shifts with experimental data, providing a robust framework for confirming the molecular structures. This approach not only substantiates the proposed structural assignments but also enhances the reliability of the configurational analysis derived from NMR spectroscopic data.

Reference

1. Lee, S.; Kim, C.S.; Yu, J.S.; Kang, H.; Yoo, M.J.; Youn, U.J.; Ryoo, R.; Bae, H.Y.; Kim, K.H. Ergopyrone, a styrylpyrone-fused steroid with a hexacyclic 6/5/6/6/6/5 skeleton from a mushroom Gymnopilus orientispectabilis. *Org. Lett.* **2021**, 23, 3315-3319, doi.org/10.1021/acs.orglett.1c00790.

2. Grimblat, N.; Zanardi, M.M.; Sarotti, A.M. Beyond DP4: An improved probability for the stereochemical assignment of isomeric compounds using quantum chemical calculations of NMR shifts. *J. Org. Chem.* **2015**, 80, 12526-12534, doi.org/10.1021/acs.joc.5b02396
